# Co-Circulation and Excretion Dynamics of Diverse *Rubula*- and Related Viruses in Egyptian Rousette Bats from South Africa

**DOI:** 10.3390/v11010037

**Published:** 2019-01-08

**Authors:** Marinda Mortlock, Muriel Dietrich, Jacqueline Weyer, Janusz T. Paweska, Wanda Markotter

**Affiliations:** 1 Department of Biochemistry, Genetics and Microbiology, Faculty of Natural and Agricultural Sciences, University of Pretoria, Pretoria 0002, South Africa; marinda.mortlock@up.ac.za; 2 Centre for Viral Zoonoses, Department of Medical Virology, School of Medicine, Faculty of Health Sciences, University of Pretoria, Pretoria 0001, South Africa; jacquelinew@nicd.ac.za (J.W.); januszp@nicd.ac.za (J.T.P.); 3 UMR Processus Infectieux en Milieu Insulaire Tropical, 97490 Sainte-Clotilde, Reunion Island, France; muriel.dietrich@gmail.com; 4 Centre for Emerging Zoonotic and Parasitic Diseases, National Institute for Communicable Diseases of the National Health Laboratory Service, Sandringham, Johannesburg 2131, South Africa

**Keywords:** paramyxovirus, rubulavirus, Egyptian rousette bat, co-circulation, viral shedding, tissue distribution, human mumps virus-related, human parainfluenza virus-related, excretion dynamics

## Abstract

The Egyptian rousette bat (*Rousettus aegyptiacus*) has previously been implicated as the natural host of a zoonotic rubulavirus; however, its association with rubulaviruses has been studied to a limited extent. Urine, spleen, and other organs collected from the *R. aegyptiacus* population within South Africa were tested with a hemi-nested RT-PCR assay targeting a partial polymerase gene region of viruses from the *Avula*- and *Rubulavirus* genera. Urine was collected over a 14-month period to study the temporal dynamics of viral excretion. Diverse rubulaviruses, including viruses related to human mumps and parainfluenza virus 2, were detected. Active excretion was identified during two peak periods coinciding with the host reproductive cycle. Analysis of additional organs indicated co-infection of individual bats with a number of different putative rubulaviruses, highlighting the limitations of using a single sample type when determining viral presence and diversity. Our findings suggest that *R. aegyptiacus* can harbor a range of *Rubula*- and related viruses, some of which are related to known human pathogens. The observed peaks in viral excretion represents potential periods of a higher risk of virus transmission and zoonotic disease spill-over.

## 1. Introduction

Bats have been shown to have a high viral richness, with a considerable proportion associated with zoonoses [1], including members from the *Paramyxoviridae* family. Paramyxoviruses are non-segmented single-stranded negative-sense RNA viruses belonging to one of seven genera with several unclassified members [2]. The emergence of the zoonotic Hendra virus in Australia and Nipah virus in Southeast Asia (*Henipavirus* genus) from pteropid bat species [3,4,5], highlighted the public and veterinary health impact of these viruses. Henipaviruses have high morbidity and mortality rates, and outbreaks are reported on a near-annual basis. The 2018 epidemic of Nipah virus reported in Kerala, India, expanded the known geographical range of the virus to southwest India [6]. A large number of putative henipaviruses have also been reported from bats sampled in several African countries [7,8,9,10]. As such, research and surveillance regarding zoonotic paramyxoviruses has mainly been targeted towards the *Henipavirus* genus. This included investigations into the diversity and distribution of the virus and host species as well as virus excretion and pathogenicity. However, the *Rubulavirus* genus also gained attention as a result of the emergence of bat-borne zoonoses.

Viruses from the *Rubulavirus* genus are known to infect humans, non-human primates, pigs, and bats [11]. Based on phylogenetic inference, the genus can loosely be divided into two clades. The first includes viruses of public and veterinary health importance such as human mumps virus (MuV), human parainfluenza virus 2 (HPIV-2), human parainfluenza virus 4 (HPIV-4), simian virus 41, and porcine rubulavirus. In addition, this clade includes a number of more recently described bat-borne viruses including Mapuera virus and putative viral species from a number of African bat species [7,12]. Mumps is a contagious disease that is spread through respiratory droplets and is mainly associated with painful swelling of the parotid and salivary glands. It is usually self-limiting [13]. It does, however, result in more severe complications in a few cases, including encephalitis, meningitis, and deafness. Although it is a vaccine-preventable disease, resurgence is observed in many locations worldwide due to anti-vaccination sentiment. HPIV-2 and 4 are the causal agents of upper and lower respiratory illnesses which can range from mild to severe outcomes but can also cause an undifferentiated febrile disease in children [14]. No vaccines for the prevention of HPIV-2 and 4 infections are currently available. The second clade consists of viruses that have only been described from bat species, with two rubulaviruses that caused disease after zoonotic events. Menangle rubulavirus emerged as a porcine pathogen in Australia and is believed to have been associated with severe flu-like symptoms in two farm workers who came into close contact with diseased pigs and presented with neutralizing antibodies to the virus [15]. The virus was subsequently demonstrated to be of bat origin following successful virus isolation from pteropid bat urine [16]. In 2014–2015, the *Rubulavirus* genus gained further attention with the report of a newly described virus, Sosuga virus, the etiological agent of a zoonotic disease spill-over from Egyptian rousette bats (*Rousettus aegyptiacus*) in Uganda, resulting in a non-fatal, yet severe, febrile disease [17,18]. The patient recovered after two-week hospitalization and remains the only case of Sosuga virus infection reported in humans. 

Despite the association of 11 of the 17 officially recognized *Rubulavirus* species [2] with various fruit bat species, distribution and prevalence studies for rubulaviruses in these mammals have been limited. Among the viruses detected are Achimota virus 1 and 2 from *Eidolon helvum* (the straw-colored fruit bat). Infection studies in a number of small laboratory animals indicated that these viruses are able to cross the species barrier, resulting in respiratory disease in ferrets [19]. In addition, a virus closely related to human MuV was detected from the fruit bat genus *Epomophorus*, genetically characterized and officially classified as the viral species *Bat mumps rubulavirus* [2,7]. Although the bat mumps virus has not been successfully isolated to date, cross-reaction and cross-neutralization with human MuV have been reported with the use of recombinant viral particles [20,21,22].

Reports on rubulavirus detection in *R. aegyptiacus* (the Egyptian rousette bat) have been limited [7]. *Rousettus aegyptiacus* is a cave-dwelling fruit bat with a wide, yet scattered, distribution across sub-Saharan Africa, but is also found in parts of North Africa, Southwest Asia, and the Middle East [23]. This bat species is a known reservoir host for other zoonotic and potentially zoonotic viruses like the Marburg virus (*Filoviridae*) [24,25,26] and the Lagos bat virus (*Rhabdoviridae*), respectively [27]. Our study aimed to expand knowledge regarding rubulaviruses and their association with a South African population of *R. aegyptiacus*. Results suggest a strong association between this host and putative rubulaviruses based on the observed diversity and co-circulation. We also detected a human MuV-related genomic sequence and a genomic sequence related to HPIV-2 and its primate counterpart simian virus 41. Tissue distribution analysis suggests that general testing using a single sample type may be biased towards certain viral species, leading to an under-estimation of the diversity within this particular bat species and under-reporting of potentially zoonotic viruses of public health interest. However, non-invasive sampling, i.e., urine collection, used in many ecological studies for paramyxoviruses, remains a viable option for general diversity studies. The latter approach was successfully used for studying *Rubula*- and related virus excretion dynamics, identifying two major peaks of viral shedding within the span of a year. 

## 2. Materials and Methods 

### 2.1. Study Site, Permits, and Ethical Statements

The study targeted an *R. aegyptiacus* maternity roost in Matlapitsi cave located in the Limpopo Province, South Africa [28], where longitudinal virological research is conducted as part of a broader biosurveillance research program on zoonotic pathogens associated with bats. The mountainous region surrounding the cave is considered predominantly rural, with local agricultural practices including citrus, vegetable, and livestock farming. Livestock (chickens, cattle, donkeys, and goats) and domestic animals are free-roaming in the area. The cave is a short hike from the main road with the entrance hidden between vegetation on the side of the mountain. The cave is frequented by the local community for traditional and religious purposes. Samples used in this study were collected over a seven-year period from 2012 to 2018. We obtained permission to conduct research under Section 20 of the Animal Disease Act (Act No. 35 of 1984) from the Department of Agriculture, Forestry and Fisheries of South Africa. For sample collection at Matlapitsi cave, a sampling permit from the Department of Economic Development, Environment and Tourism of the Limpopo Provincial Government, South Africa (CPB6-003767) was obtained. Ethical clearance for sample collection and paramyxovirus testing was provided by the University of Pretoria Animal Ethics Committee under the clearance numbers EC058-14 and EC054-14 and the National Health Laboratory Service Animal Ethics Committee with clearance number AEC-137/12. 

### 2.2. Sample Collection

During the longitudinal study, *R. aegyptiacus* sampling was conducted on a catch-and-release basis where a range of samples including blood, urine, fecal, oral, and rectal swabs were collected as part of a larger project on zoonotic pathogens in bats. In addition, as part of species diversity studies in collaboration with the Ditsong National Museum of Natural History of South Africa, voucher specimens were also collected. All sampling, voucher collection and necropsies were performed under conditions based on the risk assessment for transmission of potentially zoonotic pathogens either through direct contact or through the aerosol route. This included the use of personal protective equipment including gumboots, Tyvek^®^ coveralls, latex and leather gloves, and powered air purifying respirators. Decontamination was performed using a 10% liquid bleach (sodium hypochlorite) solution. 

Trapping at the cave entrance was done using Austbat three-bank and mini harp traps (Faunatech, Australia). Captured bats were anaesthetized using a mixture of ketamine and xylazine (1:2; a volume of 0.05 to 0.1 mg/g body mass) before bleeding, and for voucher collection, euthanasia was achieved through cardiac exsanguination. Organs were harvested during necropsy and transported in liquid nitrogen to the laboratory for storage at −80 °C. Urine was collected when available from captured bats with the use of pipettes, however, *R. aegyptiacus* rarely urinated in-hand during capturing or processing. For a more structured approach, to perform temporal analysis of viral excretion, decontaminated plastic trays treated to remove all DNase and RNases were placed within the cave underneath the roosts on a monthly basis (June 2017 to July 2018) to collect pooled off-host urine samples at the population level. Trays were placed at least one meter from each other to limit the collection of urine from the same individuals on multiple occasions, although movement of bats during sampling cannot be controlled. In addition, trays were positioned at the exact same place over the collection period. Ten urine droplets (assumed to represent ten individual bat specimens) were collected using a pipette and pooled per sample. Sample types selected for viral testing thus consisted of spleen, opportunistically collected urine samples (from individuals and pools), and targeted population-level pooled urine samples collected monthly over a continuous time period, where pool sizes and placement of trays were kept constant. For individuals where viral nucleic acids were detected in spleen tissue, additional organ tissues were tested to consider tissue distribution. A detailed list of samples collected and tested for this study is provided in Table S1. 

### 2.3. Viral PCR Screening

Approximately 30–50 mg of spleen tissue from bats were used for initial paramyxoviral RNA testing. Under biosafety level 3 (BSL3) conditions, all samples were inactivated in Trizol^®^ reagent (Invitrogen, Thermo-Fisher Scientific, Waltham, MA, USA) and homogenized in a TissueLyser II system (Qiagen, Hilden, Germany) for 45 s at 30 Hz using 5-mm stainless steel beads. Further processing and testing of samples were done under BSL2 conditions. Total RNA was extracted using the Trizol^®^ reagent (Invitrogen) according to the manufacturers’ specifications. Samples were tested with a published primer set targeting a partial polymerase gene region of the *Avula–Rubulavirus* (AR) genera with a resultant hemi-nested amplicon size of 224 bp [29]. These primers were used in combination with an in-house developed two-step hemi-nested RT-PCR assay with a sensitivity of 3 × 10^2^ RNA copies in the hemi-nested amplification round. This was determined through serial dilutions of RNA transcripts, derived from Newcastle disease virus, for which the copy number was known. Complementary DNA was generated using a SuperScript IV (ThermoFisher Scientific, Waltham, MA, USA) reverse transcription kit. For the first-round PCR amplification, a master mix was prepared by mixing 10 pmol AR Forward-F1 primer (10 pmol/µL, Integrated DNA Technologies), 15 pmol AR Reverse-R primer (10 pmol/µL, Integrated DNA Technologies), 22 mM dNTP mix (10 mM/µL, Thermo-Fisher Scientific, Waltham, MA, USA), 1× DreamTaq™ buffer (10×, Thermo-Fisher Scientific, Waltham, MA, USA), 1.25 U DreamTaq™ polymerase (5 U/µL, Thermo Scientific), and nuclease-free water (Ambion, Life Technologies, Thermo-Fisher Scientific, Waltham, MA, USA) to a final volume of 45 µL. To the master mix, 5 µL of the cDNA product were added. Amplification was performed by incubating the samples at 94 °C for 2 min; 40 cycles of 94 °C for 15 s, 48 °C for 30 s, and 72 °C for 30 s; and 72 °C for 7 min using a SimplyAmp thermocycler (Applied Biosystems, Thermo-Fisher Scientific, Waltham, MA, USA). Hemi-nested amplification was performed as described for the initial round of amplification using 15 pmol AR Forward-F2 primer (10 pmol/µL, Integrated DNA Technologies) and nuclease-free water adapted accordingly to a final volume of 47 µL. Care was taken to prevent any cross-contamination of samples by setting up reactions in dedicated biosafety level 2 cabinets, for first and nested amplification reactions. Aseptic cleaning techniques were employed to decontaminate work areas and equipment before and after use. A no-template control was included in each batch of samples tested to indicate any possible contamination of the reagents, consumables, or equipment. Hemi-nested products were analyzed on a 1.5% agarose gel. Amplicon purification was performed using the Zymoclean™ Gel DNA Recovery Kit (Zymo Research, Irvine, CA, USA) as per the manufacturers’ instructions. 

A sequencing PCR was set up using the BigDye Terminator v3.1 Cycle Sequencing Kit (Thermo Fisher Scientific) by preparing a master mix containing 1× sequencing buffer, 3.2 pmol AR Forward-F2 primer, 0.5 mM BigDye Terminator mix, and nuclease-free water (Ambion) to a final volume of 7 µL. A volume of 3 µL of purified DNA product was added to each reaction. The same master mix was prepared for the reverse reaction using the AR Reverse primer. The sequencing reactions were run at 96 °C for 1 min; and 25 cycles of 96 °C for 10 s, 50 °C for 5 s, and 60 °C for 4 min. Sequencing reactions were purified using the ethanol/EDTA/sodium acetate precipitation method as described in the BigDye Terminator v3.1 Cycle Sequencing Kit manual. Purified reactions were submitted to the ACGT DNA Sequencing facility of the University of Pretoria, South Africa, where samples were subjected to sequencing on the ABI 3500xl instrument (Thermo-Fisher Scientific, Waltham, MA, USA). For all positive individuals, other abdominal organs (where available) including kidney, liver, lung, and intestine were tested for paramyxovirus RNA as described above. Where direct sequencing proved ineffective as a result of co-infection or background amplification due to the degenerate nature of the primers, amplicons were cloned with the use of the pGEM-T Easy (Promega, Madison, WI, USA) vector system and JM109-competent cells (Promega) as per the manufacturer’s instructions, of which three or more clones were sequenced.

To obtain an extended region of the polymerase gene as well as a partial nucleoprotein (*N*) gene region for selected viruses identified in the study, the same hemi-nested RT-PCR methodology described for the *Avula–Rubulavirus* assay was applied. For the extended polymerase (*L*) gene region, AR Forward-F1, AR Forward-F2 [29], and MuV-R11334 reverse primer as per the literature [7] were used in a hemi-nested RT-PCR assay with expected amplicon sizes of 818 bp and 770 bp. For the partial nucleoprotein (*N*) gene, MuV-F648 5′-ACA GTG TVC TAA TCC AGG YTT GG-3′, MuV-F669 5′-GGR TGA TGG TCT GYA AAT GYA TGA C-3′ and MuV-R1156 5′-CAT WGC ATA RCT GAA TAT CAR TGG GTA-3′, adapted from literature [7], were used in a hemi-nested RT-PCR reaction. Expected amplicon sizes for the nucleoprotein (*N*) gene fragment were 509 bp and 488 bp.

In addition, the 90 samples positive with the *Avula–Rubulavirus* assay were tested with a broadly reactive assay specific for the *Paramyxoviridae* family (PAR) obtained from literature [29]. This was included to obtain a larger region of the polymerase (*L*) gene, to support the phylogenetic analysis of the shorted region amplified with the *Avula–Rubulavirus* assay during initial testing.

### 2.4. Bioinformatic Analysis

Prior to bioinformatics analysis, viral sequences detected in the study were aligned and sequences with a 100% amino acid identity in the analyzed region were grouped together. One representative sequence for each group was selected and included in phylogenetic analysis. All sequence alignments for phylogenetic analysis were performed in BioEdit sequence alignment editor (v7.2.5) software [30]. Where required, sequence lengths were trimmed to the same length when including representative sequences available in the public domain. To infer the best DNA substitution model for nucleotide sequence analysis, jModelTest (v2.1.10, Universidade de Vigo, Vigo, Spain) was used and MEGA5 was utilized to infer the best amino acid substitution model for the tissue distribution analysis [31]. The latter analysis was presented based on amino acid sequences to provide a more conservative comparison at functional level due to synonymous mutations in the nucleotide sequences. Bayesian phylogenetic analysis was performed in BEAST (Bayesian evolutionary analysis by sampling trees v2.5, Beast 2 development team 2011–2018) using the best fit model with 10,000,000 iterations sampling every 1000 trees (available online from http://beast.bio.ed.ac.uk). The phylogeny was generated using TreeAnnotator (part of the BEAST package) with a burn-in value of 10%, and the tree was visualized and manipulated using FigTree v1.4.2 (2006–2012 Andrew Rambaut, Institute of Evolutionary Biology, University of Edinburgh). For analysis of nucleotide and amino acid similarities, similarity plots were generated using the BioEdit sequence alignment editor (v7.2.5) software [30].

### 2.5. Temporal Analysis

Excretion data collected over a 14 month period (June 2017 to July 2018), were used to investigate the excretion dynamics using a generalized linear model (GLM) with a binomial error and a logit link function. The specific sampling date was used as an explanatory variable in the analysis. Temporal analysis was performed with the use of the R software package v.3.4.1 [32]. 

## 3. Results

### 3.1. Positivity and Co-Infections

**Figure 1:**
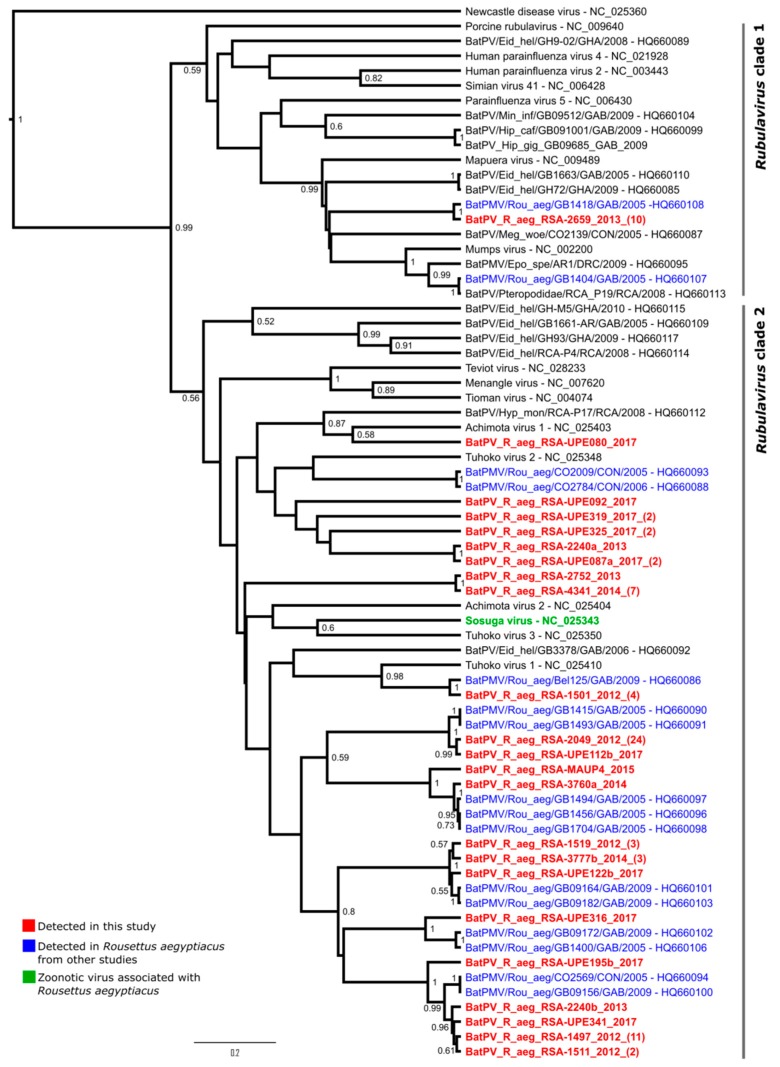
Phylogeny of a partial polymerase (*L*) gene fragment (186 nucleotides) of paramyxoviruses detected in spleen and urine samples using *Avula–Rubulavirus* genus-specific primers [29]. Bayesian phylogenetic analysis was performed using the Hasegawa-Kishino-Yano model incorporating a gamma distribution and invariant sites (HKY + I + G). Posterior probabilities of >0.5 are indicated at internal nodes. Clade 1 represents the classically known rubulaviruses infecting a range of host species, while clade 2 solely represents bat-borne *Rubula*- and related viruses. Numbers in brackets at the end of the sequence annotations for viral sequences detected in this study indicated the number of identical sequences detected based on the amino acid sequence for the target region. Newcastle disease virus (*Avulavirus* genus) was selected as the outgroup. Refer to Table 1 for accession numbers.

A total of 304 spleen samples, 58 urine samples opportunistically collected from individual bats, and 33 opportunistically collected pooled urine samples (collected for other study purposes where pools ranged from one to ten) were tested with an overall positivity of 8.6%. The latter samples were included to increase the number of urine samples to evaluate the excretion potential of these viruses via urine. With confirmation of virus excretion, a targeted longitudinal excretion analysis was performed, whereby 255 population-level pooled urine samples were tested for paramyxovirus RNA with a positivity of 15.68%. Considering the separate sample types, the rate of positivity was 9.54% for spleen, 6.89% for opportunistically collected individual bat urine samples, 3% for opportunistically collected pooled urine samples, and 15.7% for pooled population-level urine samples (collected for analysis of viral excretion dynamics). Upon analysis of the amplified partial polymerase gene region, two spleen samples (UP 3760 and UP 3777) were co-infected with two different paramyxoviruses sharing nucleotide (nt) and amino acid (aa) similarities of 74.8% and 84%, and 73.3% and 81.3% respectively. These positives are represented by sequences BatPV_R_aeg_RSA-2049_2012 and BatPV_R_aeg_RSA-3760a_2014 for UP3760, and BatPV_R_aeg_RSA-2659_2013, and BatPV_R_aeg_RSA-3777b_2014 for UP 3777 (Table 1, Figure 1). One urine sample collected from an individual bat (UP 2240), had three different viral sequences with nucleotide and amino acid similarities ranging from 74.2–80% and 80–88%, respectively, represented by BatPV_R_aeg_RSA-2049_2012, BatPV_R_aeg_RSA-2240a_2013, and BatPV_R_aeg_RSA-2240b_2012 (Table 1, Figure 1). Co-detections of two to three different viruses were also seen in pooled off-host collected urine samples. However, this may be purely due to the pooled nature of the samples and cannot be ascribed to an individual co-infection, but rather shows co-circulation of viruses within the population.

### 3.2. Phylogenetic Analysis and Identity Comparisons

**Figure A1:**
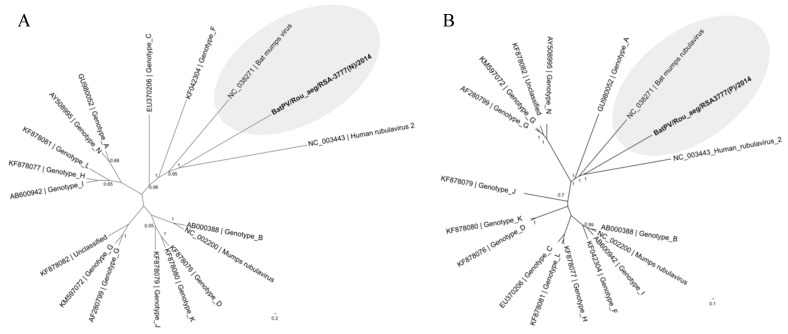
Bayesian phylogenetic analysis of two partial gene regions of a putative bat mumps rubulavirus with representatives of known mumps genotypes. (**A**) Partial nucleoprotein (*N*) gene (459 nucleotides) analysis; (**B**) Partial polymerase (*L*) gene sequence analysis (720 nucleotides). Phylogenetic analysis was performed using the Hasegawa-Kishino-Yano model incorporating a gamma distribution (HKY + G), and posterior probabilities of >0.5 are indicated at internal nodes. Sequences described from bat hosts are indicated in the grey oval shapes and the sequences detected in this study in bold type. GenBank accession numbers for sequences detected are MH883788 and MH883787, respectively.

**Figure A2:**
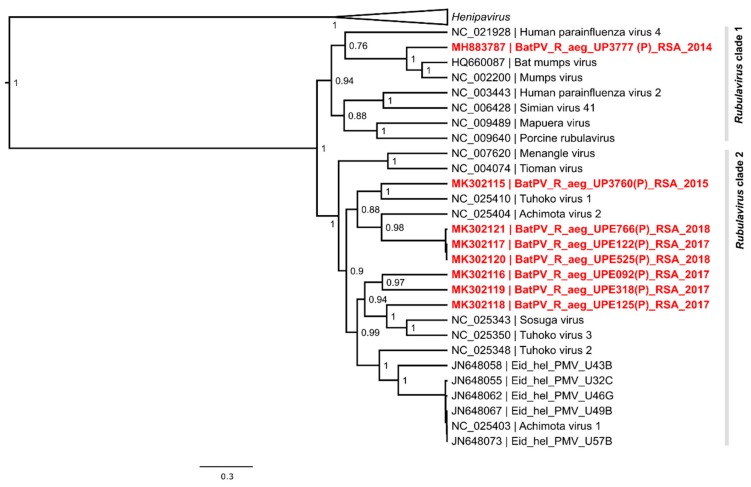
Bayesian phylogenetic analysis of a partial polymerase (*L*) gene region (529 nucleotide) of selected putative rubulaviruses detected in *Rousettus aegyptiacus*. Phylogenetic analysis was performed using the Hasegawa-Kishino-Yano model incorporating a gamma distribution (HKY + G). Sequence annotations include GenBank accession numbers.

Due to the short sequence length generated by the *Avula–Rubulavirus* assay and the necessity to trim sequences to the same length when including representative sequences found in the public domain, the posterior probabilities at internal branching points were not high enough to conclude their phylogenetic placement in relation to other classified rubulaviruses. However, sufficient support was obtained at the internal branching point separating the genus into two clades (Figure 1). Within the first clade, a putative human mumps-related virus (represented by BatPV_R_aeg_RSA-2659_2013) was detected on multiple occasions, i.e., in seven spleen samples and three urine pools (Table 1), and grouped phylogenetically close to a viral sequence (BatPV/Rou_aeg/GB1418/ GAB/2005) described from the same species sampled in Gabon in 2005 as well as the previously described bat mumps virus from the *Epomophorus* fruit bat genus (BatPMV/Epo_spe/AR1/DRC/2009). To further support this grouping with human mumps virus, an extended polymerase gene region and partial nucleoprotein region were obtained from the detected *R. aegyptiacus*-associated putative bat mumps virus in this study. Analysis of these gene regions grouped this virus with other human mumps virus genotypes as well as the bat mumps virus described from *Epomophorus* sp., with high posterior probabilities (Figure A1). Although an overall low phylogenetic resolution was observed within *Rubulavirus* clade 2, the majority of sequences described in this study grouped in this clade along with other fully characterized and officially classified bat-borne rubulavirus species, i.e., Sosuga virus, Achimota virus 1 and 2, Tuhoko virus 1, 2 and 3, Menangle virus, and Tioman virus. The grouping within this clade is, however, in line with findings from another study [7] where a higher phylogenetic resolution was obtained due to the availability of first-round amplicons of a larger size. A large proportion of the detected viral sequences was grouped closely with viral sequences detected in *R. aegyptiacus* sampled in Gabon and Congo in previous years, potentially indicating a species specificity of these viruses which may not be affected by the geographical distribution, supporting previous suggestions of co-evolution between bats and paramyxoviruses [7]. The amplification of larger gene regions of the polymerase (*L*) gene of the viral sequences detected during initial testing was only achievable with a number of positive samples. Nonetheless, phylogenetic analysis of these sequences supports the overall groupings of the viral sequences within *Rubulavirus* clade 1 and clade 2 (Figure A2). 

Nucleotide and amino acid similarity comparisons to fully characterized *Rubulavirus* species indicated that the representative sequence BatPV_R_aeg_RSA-2659_2013 shared a 76.8% nucleotide similarity and a high amino acid similarity of 96.7% (two amino acid changes within the analyzed region) with human mumps virus (Table 1). Similar identities were seen with the partial nucleoprotein and extended polymerase region upon analyses. The observed difference in similarities in nucleotide and amino acid levels of the partial polymerase gene region proved to be as a result of a high proportion of synonymous substitutional differences between these sequences. A similar observation was made for other sequences including BatPV_R_aeg_RSA-3777b_2014, where more than a 10% difference in similarity at the nucleotide and amino acid level was observed. The sequence was most similar at the nucleotide level to Tuhoko virus 1, a *Rousettus leschenaultia*-derived bat sequence from China, but on an amino acid level it was more similar to Achimota virus 2, isolated from *E. helvum* bats in Africa, as a result of a high number of synonymous mutations in the third codon position. Considering all sequences grouping within the *Rubulavirus* clade 2 (Figure 1), the majority is related to the Achimota and Tuhoko viruses at the nucleotide level while a considerable proportion is closely related to the *R. aegyptiacus*-associated Sosuga virus at the amino acid level (Table 1).

### 3.3. Tissue Distribution

**Figure 2:**
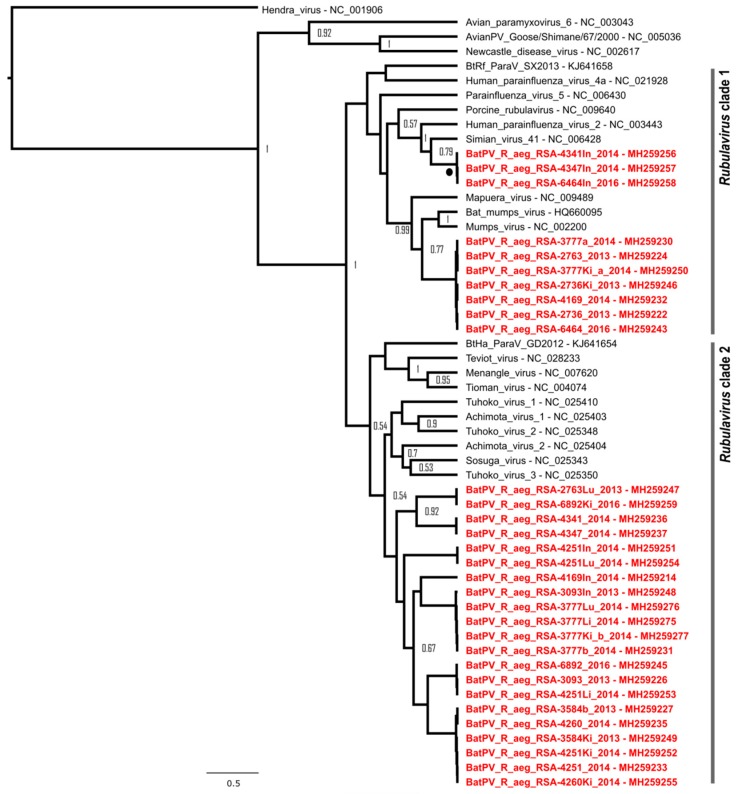
Phylogeny of a partial polymerase (*L*) gene fragment (210 nucleotides) of paramyxoviruses detected in organ tissue using *Avula–Rubulavirus* genus-specific primers. Bayesian phylogenetic analysis was performed using the TPM3uf model incorporating a gamma distribution (TPM3uf + G). Posterior probabilities of >0.5 are indicated at internal nodes. Sequences indicated in red represent viral sequences detected from organ tissues of individuals that were positive in initial spleen testing. Sequence annotations include abbreviations for additional organs (Ki: kidney, Li: liver, Lu: lung, In: intestines) as well as accession numbers. The black circle indicates an additional sub-clade detected in intestinal tissue not previously detected during spleen and urine testing.

To investigate the within-host distribution of the detected *Rubula*- and related viruses, two or more additional organs available for 27 of the 29 spleen-positive bats were tested. Twelve of the animals proved to have at least one additional organ positive (Table 2). Notably, two samples, UP 3777 and UP 4251, were positive in three and four of the four additional organs, respectively, and the former was co-infected with two paramyxoviruses in the kidney tissue. Upon phylogenetic analysis of nucleotide sequences, the topological profile observed was similar to that of the initial detection results, with a mumps-related clade (*Rubulavirus* clade 1) and a bat-specific clade (*Rubulavirus* clade 2) (Figure 2). However, an additional clade was detected within the larger *Rubulavirus* clade 1, which was related to human parainfluenza virus-2 and simian virus 41, with a high posterior probability. Based on phylogenetic placement within the clade, human parainfluenza virus-2 seems to be ancestral to the bat-derived putative parainfluenza virus. Interestingly, viral RNA representing the putative bat parainfluenza virus was only detected in intestinal tissue. 

**Figure 3:**
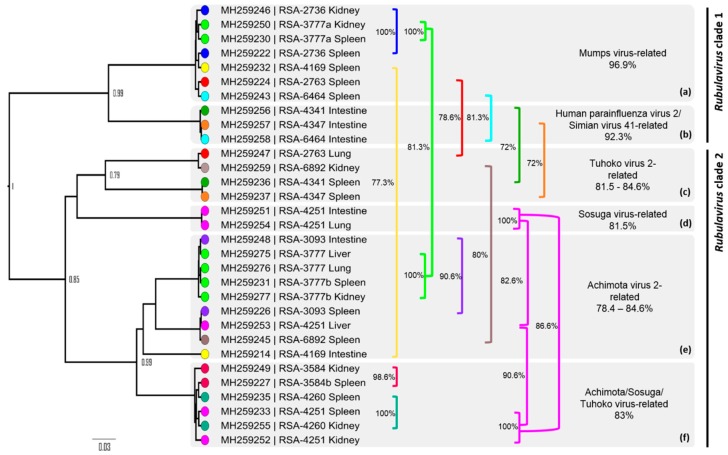
Phylogeny of a 75-amino acid length fragment of the polymerase (*L*) gene of paramyxoviruses detected in various tissues using *Avula–Rubulavirus* genus-specific primers. Bayesian phylogenetic analysis was performed using the WAG model with a gamma distribution (WAG + G). Organs are color-coded per bat and the amino acid similarity between viral sequences detected per individual bat is indicated in the corresponding colored bracket. Greyed blocks labelled with alphabetical letters indicate sub-clades within the larger two *Rubulavirus* clades and the highest amino acid similarity per sub-clade to fully characterized paramyxoviruses recognized by the International Committee on Taxonomy of Viruses (ICTV; available online at https://talk.ictvonline.org/taxonomy/). Sequence annotations include accession numbers, location, laboratory number, and organ.

Nucleotide and amino acid analysis indicated that viral sequences detected in kidney tissue were, in most cases, identical to those detected in spleen samples during initial detection (Figure 3). For sample UP 3777, two genetically diverse viruses were co-infecting the spleen tissue, of which both were also observed in the kidney. For other organs, however, bats were co-infected with viruses belonging to different sub-clades within the genus (Figure 3a–f). Viruses related to human mumps and human parainfluenza virus-2 had the highest similarity to previously classified rubulaviruses, with amino acid similarities of 96.9% and 92.3%, respectively. Sequences in the bat-specific clade (*Rubulavirus* clade 2) were more diversified, with amino acid similarities ranging from 78.4% to 84.6% in comparison to previously described bat rubulaviruses. Two smaller clades (Figure 3d,f) were 81.5% and 83% similar to the zoonotic Sosuga virus at the amino acid level. The former was detected in intestinal and lung tissue, whereas the latter was present in kidney and spleen tissue. 

### 3.4. Temporal Analysis

**Figure 4:**
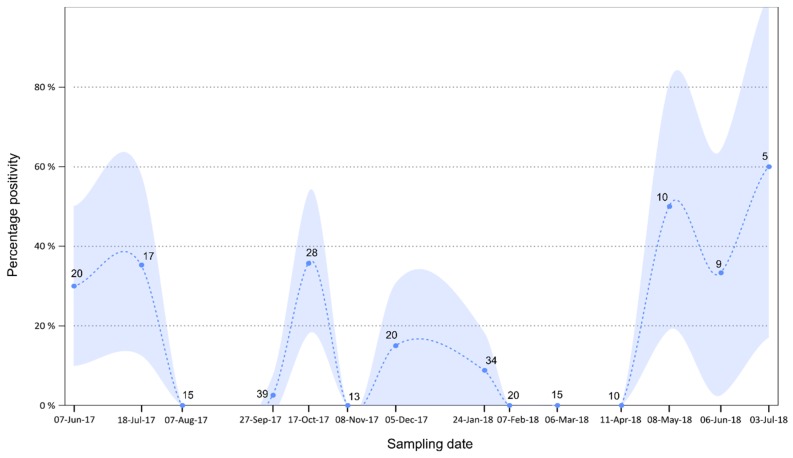
Temporal dynamics of rubulavirus excretion in urine within a *Rousettus aegyptiacus* population in South Africa detected with the *Avula–Rubulavirus*-specific primers [29]. Raw data are indicated by circles, and the dashed line shows values predicted by a loess function in R software [32]. Shaded areas represent 95% confidence intervals.

A total of 255 targeted population-level pooled urine samples collected longitudinally over 14 months were molecularly tested to determine their positivity for paramyxovirus RNA. We found an overall positivity rate of 16% (±4%) and a significant variation over time (χ^2^_14_ = 59.999, *p* < 0.001; Figure 4). The excretion dynamic was characterized by two major peaks in June–July (35 ± 20% in 2017) and October (35% ± 18%). The excretion dropped to zero in November but this might be due to a limited number of samples (*n* = 13) at this date. Excretion remained high in December and January and stabilized to zero through to May, where positivity again peaked and reached a maximum in July 2018 (60% ± 43%).

## 4. Discussion

Information regarding the association of rubulaviruses and bats have been limited, even more so for the fruit bat species *R. aegyptiacus*. With the detection of a zoonotic rubulavirus, Sosuga virus, from this species [18], more research is needed regarding this widely distributed African bat species as a natural host to paramyxoviruses. Our study was able to obtain more information on the association between rubulaviruses and an *R. aegyptiacus* population in South Africa.

### 4.1. Detection of Nucleic Acids

A number of putative *Rubula*- and related virus RNA was detected in spleen and urine. When considering the overall positivity observed from spleen tissue (9.54%), our results are comparable to those of another organ-targeted study reporting a positivity of 7.04% from the same species sampled in Ghana, Gabon, the Democratic Republic of Congo, and Congo-Brazzaville, collectively (Table 3) [7]. Although the use of spleen provided data on a number of these viruses, off-host collected urine samples proved to be even more effective at detecting a diversity of viruses from these bats at specific times of the year. Based on these findings, urine collection provided a robust non-destructive method to study rubulavirus diversity in bats. 

The detection of a virus closely related to human mumps virus in both spleen tissue and urine suggests urine as a possible route of transmission. Given its close relatedness to human mumps virus, oral excretion of this virus cannot be excluded and warrants additional research. A recent study reported on the detection of both *Rubulavirus*-related as well as unclassified paramyxoviruses in pharyngeal and anal swabs collected from insectivorous bats with the use of next-generation sequencing, implicating these as additional routes of potential paramyxovirus transmission [34]. It has been suggested that the presence of an animal reservoir for a virus conspecific to human mumps virus could influence current human disease control strategies [7]. Studies using recombinant human mumps virus particles with a human mumps backbone containing the fusion (F) and hemagglutinin-neuraminidase (HN) surface proteins of bat mumps virus reported on both cross-reaction and cross-neutralization of these viruses in cell culture [20,21,22]. As such, vaccination of the human population against human mumps virus likely provides protective immunity against these bat-borne counterparts. 

The detection of nearly identical sequences, as well as the clustering of viral sequences detected in *R. aegyptiacus* from South Africa and other distant African countries, could be indicative of the co-evolution of *Rubula*- and related viruses and this natural host species. This is additionally supported by the geographical distances between these bat populations as *R. aegyptiacus* has to date only been recorded to travel 500 km at the most [28]. Information regarding migration of this species is limited and will be required to determine whether these bats form part of a metapopulation. However, none of the viruses described in this study were as closely related to Sosuga virus on nucleotide level as observed for the aforementioned examples. Amino acid analysis of viruses detected in South African rousettes, do however indicate that they might be more similar to Sosuga virus on a functional level. Nonetheless, although a high prevalence of up to 10.25% for this virus was previously reported in Ugandan bat populations with the use of a highly sensitive qRT-PCR assay specifically targeting the nucleoprotein gene of Sosuga virus [18], our data suggests that this zoonotic virus is not currently circulating within this specific South African *R. aegyptiacus* population or perhaps is below the detection threshold of our current assay. 

The application of a degenerate broadly reactive nucleic acid detection assay for the current diversity study has allowed for the detection of different *Rubula*- and related viruses from *R. aegyptiacus*. This assay was selected instead of the *Paramyxoviridae*-specific (PAR) assay for general detection studies due to the differences in assay sensitivity and specificity [29]. The data obtained from using the *Avula-–Rubulavirus* assay is, however, limited in sequence length and allows for only limited phylogenetic analysis. Low posterior probabilities (<0.5) allowed only for conclusions on the relatedness of sequences to other viral sequences and genera. No additional phylogenetic inferences and conclusions could be made. Although this assay is useful for detection of diverse *Rubula*- and related viruses, there remains a need for updated assays which can be used for further characterization of these viruses. Although the *Paramyxovirus*-specific assay was able to provide additional genetic information on a small number of the viral sequences detected, these extended sequences could not be compared to other viral sequences previously described from *R. aegyptiacus* as sequence information for this region is not available. Previous attempts at virus isolation in Vero cell culture, and the application of next-generation Illumina sequencing from biological samples, was not successful. As such, additional genome information could not be obtained from these viruses. 

### 4.2. Tissue Distribution

By analyzing the tissue distribution of the paramyxoviruses, we additionally detected a considerable number of co-infections within the different organs of individual bats (Figure 3). This was also observed in single spleen and urine samples. This provided insight into the limitation of using a single sample type when studying the diversity of paramyxoviruses within this and potentially other host species, and also highlights the wide tissue distribution of paramyxoviruses. However, spleen tissues proved to be useful as a target sample type for organ-based detection of *Rubula*- and related viruses in fruit bats as previously described for paramyxoviruses in *R. aegyptiacus* and a number of other fruit bat species [7]. This was supported by the detection of paramyxovirus RNA in the spleen, but in no other organs for several individuals. It can be hypothesized that viruses remain latent or replicate at low levels within splenic tissue and, during times of increased stress, increase replication and spread to other organs. 

The diversity of paramyxovirus sequences detected was also reported in spleen tissue, except in the case of intestinal samples. As a result of the small sample size of organs additionally tested, no conclusions can be drawn on the potential tropism of these viruses. The observed distribution of the viruses in the various tissues might be a result of differences in RNA concentrations within the tissues. Future application of quantitative real-time assays and inclusion of a larger sample size would provide more insight into this. 

In addition to identifying a considerable number of co-infections in individual bats, our research identified a putative virus species closely related to human parainfluenza virus-2 and its primate counterpart simian virus 41, with a potential tropism for the intestinal tissue. Simian virus 41 was initially described from a cynomolgus monkey (*Macaca fascicularis*) [35] which has, in recent years, been documented to predate on bats in Kenya and Tanzania [36]. Given the seemingly close phylogenetic relationship between simian virus 41, human parainfluenza virus 2, and the partial bat parainfluenza virus sequence detected in this study (BatPV_R_aeg_RSA-4341In_2014, accession number MH259256), it could be hypothesized that interspecies transmission and subsequent host adaptation could have taken place. 

In Africa, such a scenario is plausible given the observed niche overlap between humans, primates, and bats. In addition to the predation on bats by primates, bush meat is a protein source in many African countries [37]. Hunting for and butchering of bush meat results in close contact between wildlife and human population and this has been suggested as a risk factor for exposure to potentially zoonotic bat-borne henipaviruses in Africa [38]. The overlapping habitat between these three populations provide increased exposure and potential for virus transmission. Concurrently, partially eaten fruit contaminated with bat excretions (i.e., saliva, urine, or feces) could also provide a means of virus exposure and transmission, as has been shown for Nipah virus (*Henipavirus* genus), a bat-borne paramyxovirus which has been successfully isolated from swabs taken off partially eaten fruit [39]. Exposure of the bats to primate excreta or partially eaten fruit infected with primate-borne viruses cannot be excluded due to the shared habitat and will require more research. Our study did not evaluate oral and fecal routes of virus transmission, which might provide future insight on the potential routes of transmission of these parainfluenza- and other related viruses. 

With the current data from this study, we have limited genomic information on the putative bat parainfluenza virus which places the virus basal to both its human and primate counterparts, suggesting human parainfluenza virus 2 as the ancestral virus to the clade. Such an observation could be indicative of reverse zoonotic disease transmission (zooanthroponosis). Nonetheless, potential human infection with a bat-borne parainfluenza virus might have gone undetected or misdiagnosed given its general association with respiratory and undifferentiated febrile disease. Previous serological findings of human infections with simian virus 41 [35] may have been due to cross-reaction from a different closely related bat-borne virus. This hypothesis will require more research and, as such, full genome analysis and isolation will be imminent, allowing for a more comprehensive genomic analysis to determine its phylogenetic placement in relation to the abovementioned viruses as well as analysis of cross-reaction and cross-neutralization with other closely related viruses. 

### 4.3. Excretion Dynamics

The sampling site in this study is characterized by fluctuation in colony size throughout the year [28]. A peak in colony size is observed over the breeding season (September to January), while it is at its lowest during the winter months (June to August). With the availability of longitudinal sample collection from this *R. aegyptiacus* population, we were able to gain more knowledge regarding the excretion dynamics across seasons and during the breeding period of this species. 

**Figure A3:**
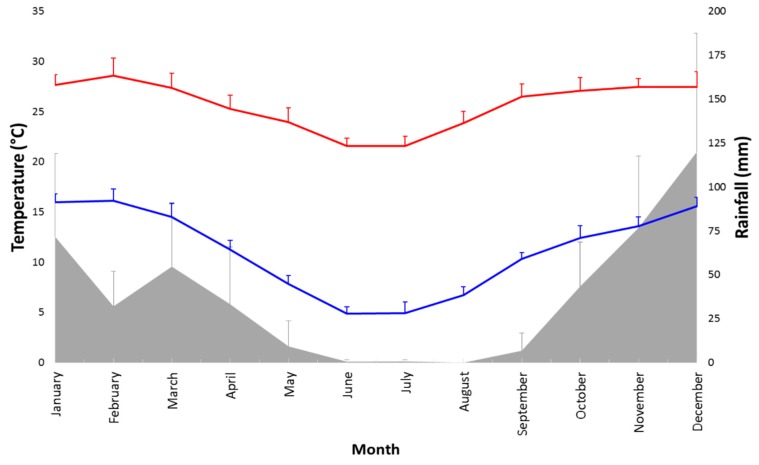
Monthly temperature and rainfall data for the study area averaged over a six-year period from 2012–2017. The red line and data points indicate maximum monthly temperatures, the blue line and data points represents minimum temperatures, and the shaded grey area indicates average monthly rainfall. Error bars indicate the standard deviation per month between the years. (Weather data provided by the South African Weather Service).

The peak observed in October coincides with the period when females aggregate within the cave and are in the late stages of gestation. Moreover, during lactation (December to January), a high percentage of virus excretion is still evident. Our results thus support a general pattern where seasonal aggregation and female reproduction favor paramyxovirus transmission [40,41]. Then, the second excretion peak observed in June–July coincides with the presumed waning of maternal antibodies as demonstrated for Marburg virus in the same bat population [26]. This results in an influx of naïve individuals into the population susceptible to virus transmission followed by increased virus replication and excretion in newly infected individuals. This is also the start of the winter period for the region, characterized by little to no rainfall, cold temperatures (Figure A3), and decreased food availability [28]. Although other less nutritional food sources might be available, the main food source for *R. aegyptiacus,* i.e., fruit from trees in the *Ficus* genus, is limited during this time. This could potentially place bats that remain in the roost over this period under nutritional stress [28] leading to an increase in virus replication and subsequent excretion. In addition, aggregation of the bats around limited food sources could allow for closer contact and intraspecies transmission of these viruses during this time. Although this has not previously been studied for bat-associated rubulaviruses, nutritional stress has been documented as a factor affecting the magnitude of Hendra virus excretion, a bat-associated paramyxovirus from the *Henipavirus* genus [40]. 

Although these factors might contribute to the observed peaks in virus excretion during these periods, the collection of these samples at population levels does not allow for precise conclusions regarding all contributing factors. Targeted individual sampling during these periods of higher excretion would provide more information on the age and sex of bats implicated during each peak. Nonetheless, the temporal findings we provide are novel regarding bat-associated rubulaviruses. In addition, we were able to identify periods of higher risk of virus transmission and potential spill-over to other susceptible host species. Given differences in the ecology of *R. aegyptiacus* in South Africa when compared to more centrally located African countries, i.e., environmental conditions as well as reproductive differences, these findings might not be the same for the different regions. Should the excretion dynamics be related to reproduction, countries such as Uganda, where bimodal birthing pulses are observed in *R. aegyptiacus* [42], might have more periods of peak viral excretion if not continuous excretion due to the short succession between birthing periods. In addition, the more tropical climate in these areas, characterized by abundant rainfall and no distinct winter period (as is the case for our sampling region in South Africa), allows for the availability of food all year round and would potentially not have nutritional stress as a driving factor for virus excretion. Temperature and rainfall patterns within South Africa also differ between regions, where a winter rainfall period is evident in the Western Cape Province as opposed to the summer rainfall period for our sampling site. Studying *R. aegyptiacus* populations from other areas might shed light on the effect of climate differences on rubulavirus excretion. 

## 5. Conclusions

Our research has provided preliminary information on the association of *R. aegyptiacus* and rubulaviruses with regards to diversity, tissue distribution, excretion, and co-circulation. To our knowledge, we provide the first temporal data of longitudinal rubulavirus excretion dynamics from a wild bat population—identifying two periods with a high risk of virus transmission and potential spill-over. Spleen and urine samples proved to be ideal for detection of viral nucleic acids, while the inclusion of other sample types provided more insight into diversity, as certain paramyxoviruses might have more specific tissue tropisms. Although a low rate of positivity for paramyxoviruses has previously been reported from fecal, rectal, and intestinal samples, these sample types should not be disregarded, as potential zoonotic viruses might go undetected, as is the case with the putative bat parainfluenza virus. In addition, quantitative real-time PCR analysis will in the future greatly contribute to our understanding of tissue tropism and tissue distribution. Our data not only provided useful information for the local bat population but could be extrapolated to other populations within Africa where exposure of humans to these bat populations is more pronounced.

## Figures and Tables

**Table 1:** Representative sequences for the multiple rubulaviruses detected and partial polymerase gene sequence identity comparison between viral sequences detected in *Rousettus aegyptiacus* and officially characterized *Rubulavirus* species.

Representative Virus Sequence ^†^ (Number of Detections)	Samples Positive (Sample Type) *^,^^	GenBank Accession Numbers	Highest Similarity (%) to Classified *Rubulavirus* Species ^#^			
			Virus	nt	Virus	aa
BatPV_R_aeg_RSA-1497_2012 (11)	UP 1497 (S), UP 3584 (S), UP 4251 (S), UP 4260 (S), UP 4488 (U), UP 5261 (U),UPE 091 (U), UPE 326 (U), UPE 337 (U), UPE 479 (U), UPE 489 (U)	MH259215; MH259227; MH259233; MH259235; MH259263; MH259264; MH259270; MH259289; MH259292; MH259296; MH259298	AchPV-1	76.3%	AchPV-1, AchPV-2, ThkPV-1, ThkPV-2, Sosuga	82.2%
BatPV_R_aeg_RSA-1501_2012 (4)	UP 1501 (S) UP 2729 (S), UP 5862 (S),UPE 094 (U)	MH259216; MH259221; MH259240; MH259272	ThkPV-1	78.4%	ThkPV-1	83.8%
BatPV_R_aeg_RSA-1511_2012 (2)	UP 1511 (S), UPE 816 (U)	MH259217; MH937577	AchPV-2	76.3%	AchPV-2	83.8%
BatPV_R_aeg_RSA-1519_2012 (3)	UP 1519 (S), UP 3011 (S), UP 4729 (S)	MH259218; MH259225; MH259238	ThkPV-1	72%	AchPV-2, TioPV	80.6%
BatPV_R_aeg_RSA-2049_2012 (24)	UP 2049 (S), UP 2240 (U), UP 3093 (S), UP 3760 (S), UP 5119 (S), UP 5908 (S), UP 6101 (S), UP 6892 (S), UP 6910 (S), UPE 088 (U), UPE 113 (U), UPE 117 (U), UPE 195 (U), UPE 337 (U), UPE 527 (U), UPE 529 (U), UPE 761 (U), UPE 762 (U), UPE 764 (U), UPE 766 (U), UPE 769 (U), UPE 789 (U), UPE 808 (U), UPE 813 (U)	MH259219; MH259262; MH259226; MH259229; MH259239; MH259241; MH259242; MH259245; MH259265; MH259269; MH259274; MH259278; MH259284; MH259293; MH259300; MH259301; MH937567; MH937568; MH937569; MH937570; MH937571; MH937572; MH937573; MH937575	MenPV	74.1%	AchPV-2	82.2%
BatPV_R_aeg_RSA-2240a_2013 (1)	UP 2240 (U)	MH259260	ThkPV-2	75.8%	Sosuga	79%
BatPV_R_aeg_RSA-2240b_2013 (1)	UP 2240 (U)	MH259261	AchPV-2	74.7%	AchPV-1, AchPV-2, ThkPV-1, ThkPV-2, Sosuga	79%
BatPV_R_aeg_RSA-2659_2013 (10)	UP 2659 (S), UP 2736 (S), UP 2763 (S), UP 3777 (S), UP 4169 (S), UP 6464 (S), UP 6469 (S), UPE 525 (U), UPE 809 (U), UPE 815 (U)	MH259220; MH259222; MH259224; MH259230; MH259232; MH259243; MH259244; MH259299; MH937574; MH937576	MuV	76.8%	MuV	96.9%
BatPV_R_aeg_RSA-2752_2013 (1)	UP 2752 (S)	MH259223	ThkPV-1	70.9%	ThkPV-2	79%
BatPV_R_aeg_RSA-3760a_2014 (1)	UP 3760 (S)	MH259228	ThkPV-1, AchPV-1	70.4%	AchPV-2, Sosuga	75.8%
BatPV_R_aeg_RSA-3777b_2014 (3)	UP 3777 (S), UP 4252 (S), UPE 087 (U)	MH259231; MH259234; MH259268	ThkPV-1	73.6%	AchPV-2	83.8%
BatPV_R_aeg_RSA-4341_2014 (7)	UP 4341 (S), UP 4347 (S), UPE 118 (U), UPE 119 (U), UPE 318 (U), UPE 331 (U), UPE 343 (U)	MH259236; MH259237; MH259279; MH259280; MH259286; MH259291; MH259295	AchPV-1	70.9%	ThkPV-2	80.6%
BatPV_R_aeg_RSA-MAUP4_2015 (1)	MaUP4 (U)	MH259213	ThkPV-2	70.9%	AchPV-2, ThkPV-2, Sosuga	75.8%
BatPV_R_aeg_RSA-UPE080_2017 (1)	UPE 080 (U)	MH259266	ThkPV-2	76.3%	AchPV-1	83.8%
BatPV_R_aeg_RSA-UPE087a_2017 (2)	UPE 087 (U), UPE 195 (U)	MH259267; MH259282	ThkPV-2	78.4%	Sosuga	80.6%
BatPV_R_aeg_RSA-UPE092_2017 (1)	UPE 092 (U)	MH259271	HPIV-4a	72.5%	Sosuga	82.2%
BatPV_R_aeg_RSA-UPE112b_2017 (1)	UPE 112 (U)	MH259273	MenPV, ThkPV-1	72.5%	AchPV-2, Sosuga	80.6%
BatPV_R_aeg_RSA-UPE122b_2017 (1)	UPE 122 (U)	MH259281	ThkPV-1	73.6%	AchPV-2	85.4%
BatPV_R_aeg_RSA-UPE195b_2017 (1)	UPE 195 (U)	MH259283	AchPV-2	75.2%	Sosuga	82.2%
BatPV_R_aeg_RSA-UPE316_2017 (1)	UPE 316 (U)	MH259285	AchPV-2	75.8%	Sosuga	80.6%
BatPV_R_aeg_RSA-UPE319_2017 (2)	UPE 319 (U), UPE 481 (U)	MH259287; MH259297	AchPV-1	76.3%	AchPV-1s	85.4%
BatPV_R_aeg_RSA-UPE325_2017 (2)	UPE 325 (U), UPE 327 (U)	MH259288; MH259290	ThkPV-2	76.3%	ThkPV-1, ThkPV-2	85.4%
BatPV_R_aeg_RSA-UPE341_2017 (1)	UPE 341 (U)	MH259294	AchPV-2	76.8%	AchPV-1, AchPV-2, ThkPV-1, ThkPV-2, Sosuga	80.6%

^†^ Sequence annotation represented by: Bat paramyxovirus (BatPV), host species (R_aeg), location (RSA), laboratory number and sampling year. * Sample type abbreviated as (S) for spleen and (U) for urine. ^ Sequences with a 100% amino acid identity in the analyzed region to the representative sequence. ^#^ Species abbreviations according to the 2018 update for *Mononegavirales* classification [33]; nucleotide (nt) and amino acid (aa) similarities based on the partial polymerase gene region amplified (186 nt) using the *Avula–Rubulavirus* assay also used for phylogenetic analysis (Figure 1).

**Table 2:** Paramyxovirus RNA detection in additional organs of bats that tested rubulavirus-positive in spleen tissue.

Sample	Collection Date	Li	Ki	Lu	Int	GenBank Accession Numbers
UP 2736	July 2013	−	+	−	−	MH259246
UP 2763	July 2013	−	−	+	−	MH259247
UP 3093	September 2013	−	−	−	+	MH259248
UP 3584	November 2013	−	+	−	−	MH259249
UP 3777 *	June 2014	+	+	+	−	MH259275; MH259250; MH259276; MH259277
UP 4169	May 2014	−	−	−	+	MH259214
UP 4251	June 2014	+	+	+	+	MH259251; MH259254; MH259253; MH259252
UP 4260	June 2014	−	+	−	−	MH259255
UP 4341	July 2014	−	−	−	+	MH259256;
UP 4347	July 2014	−	−	−	+	MH259257
UP 6464	April 2014	−	−	−	+	MH259258
UP 6892	June 2016	−	+	−	−	MH259259

* Kidney co-infected with two paramyxoviruses. Li: Liver; Ki: Kidney; Lu: Lung; Int: Intestine. Plus sign (+): paramyxovirus positive; minus sign (−): paramyxovirus negative.

**Table 3:** A summary of paramyxovirus detection through molecular detection studies and percentage positivity in *Rousettus aegyptiacus*.

Reference	Tested (Positive)	Positivity	Sample Type	Countries ^#,^^
Sosuga virus prevalence studies (qRT-PCR targeting the nucleoprotein of Sosuga virus)				
Amman et al., 2015 [18]	122 (3)	2.46%	Liver/Spleen	**Uganda** (various caves within the country with varying positivity)
	401 (3)	0.75%		
	408 (15)	3.68%		
	400 (41)	10.25%		
*Rubula*- and related virus detection studies (*Avula–Rubulavirus* specific RT-PCR assay targeting the polymerase gene)				
Drexler et al., 2012 [7]	213 (15)	7.04%	Spleen	Ghana, **Gabon**, DRC, **Congo**
Current study	304 (29)	9.54%	Spleen	**South Africa**
	58 (4)	6.89%	Urine *	

* Data from individually sampled bats only. ^#^ DRC: Democratic Republic of the Congo; Congo: Republic of the Congo (Congo-Brazzaville). ^ Bold type indicates countries where positive bats were detected.

## Supplementary Materials

The following are available online at http://www.mdpi.com/1999-4915/11/1/37/s1, Table S1: Details on all samples tested for paramyxovirus prevalence, tissue distribution and excretion.
